# Effect of Long-Term Climbing Training on Cerebellar Ataxia: A Case Series

**DOI:** 10.1155/2011/525879

**Published:** 2011-11-24

**Authors:** Stephan Marianne Anke, Krattinger Sylvie, Pasquier Jérôme, Bashir Shahid, Fournier Thomas, Ruegg Dieter Georg, Diserens Karin

**Affiliations:** ^1^Department of Medicine, University of Fribourg, 1700 Fribourg, Switzerland; ^2^Neurological Center Plein Soleil, 1010 Lausanne, Switzerland; ^3^Department of Mathematics, University of Fribourg, 1700 Fribourg, Switzerland; ^4^Unit of Early Neurorehabilitation, Department of Clinical Neurosciences, University Hospital, 1011 Lausanne, Switzerland

## Abstract

*Background*. Efficient therapy for both limb and gait ataxia is required. Climbing, a complex task for the whole motor system involving balance, body stabilization, and the simultaneous coordination of all 4 limbs, may have therapeutic potential. *Objective*. To investigate whether long-term climbing training improves motor function in patients with cerebellar ataxia. *Methods*. Four patients suffering from limb and gait ataxia underwent a 6-week climbing training. Its effect on ataxia was evaluated with validated clinical balance and manual dexterity tests and with a kinematic analysis of multijoint arm and leg pointing movements. *Results*. The patients increased their movement velocity and achieved a more symmetric movement speed profile in both arm and leg pointing movements. Furthermore, the 2 patients who suffered the most from gait ataxia improved their balance and 2 of the 4 patients improved manual dexterity. *Conclusion*. Climbing training has the potential to serve as a new rehabilitation method for patients with upper and lower limb ataxia.

## 1. Introduction

Besides its known main motor symptoms (ataxia, dysmetria, dysdiadochokinesis, and intention tremor), the impact of cerebellar damage on motor learning is still unclear. For example, visuomotor adaptation [1] and the learning of multijoint figure 8 movements in the air have been shown to be impaired [2]. In contrast, patients with cerebellar lesions have been able to improve their performance in a series of 2-dimensional tracing tasks [3, 4], to adapt their gait pattern in response to changes in treadmill speed [5] and to improve postural stability in the course of a 6-week balance training [6]. Overall, it is thus unknown to what extent and under which conditions motor learning and the rehabilitation of motor function are possible in cerebellar patients. Treatment of ataxia is one of the most difficult challenges of neurorehabilitation. Pharmacotherapies are unrewarding and data on neurosurgery or rehabilitation have shown some promising results, but are insufficient to lead to any practical recommendations [7]. Up to now, there is no recognized and efficient treatment available, and different facilitation or task-oriented therapeutical techniques are applied [8]. 

Preliminary data suggested that climbing could be suitable to improve motor function in cerebellar patients (master thesis, Damien Currat and Jean-Philippe Bassin 1994, Switzerland). Climbing is a complex task for the whole motor system involving balance, body stabilization, and the simultaneous coordination of all 4 limbs. Climbing grips vary in form and distance and provide only small supporting areas for feet and hands, which compels the climber into a variety of different body positions. The climber has to repeatedly shift his/her body weight in order to stabilize his/her trunk to reach the adequate grips. Furthermore, climbing requires precise reaching movements of hands and feet to be able to hold the grips.

Currently, there are several ways of measuring ataxia, ranging from simple functional tests such as timed walks, target board tests, writing and drawing tests, and measuring the volume of water spilt from a cup [9, 10], to rating scales such as the Kurtzke functional system scale [11]. However, it has been suggested that a kinematic analysis of reaching movements is more sensitive in detecting small changes in motor function than the available clinical tests [12]. Moreover, restoration of motor function is a long-term process and ataxia affects upper as well as lower limb movements. Most studies, however, have investigated short-term motor learning within one single-training session with usually only the upper limbs involved [1–5].

The aim of the present study was therefore to evaluate the effect of a long-term 6-week climbing training on cerebellar ataxia with validated clinical balance and manual dexterity tests, and with a kinematic analysis of multijoint pointing movements of the upper and lower limbs.

## 2. Methods

### 2.1. Subjects

Participants with cerebellar ataxia were recruited from an outpatient's service of a neurorehabilitation center. We identified 4 right-handed males (22, 29, 42, and 56 years old) who met our inclusion criteria: a definite diagnosis of cerebellar ataxia, the minimal ability to maintain the standing position with help (Functional Ambulatory Category [FAC] > 0) [13], and no acute or progressive neurological diagnosis. These patients were interested in participating in our study and gave their written informed consent. The study was conducted in accordance with the standards of the institutional ethics committee. The patients were diverse in terms of pathology, duration of illness, and age, but in all of them the brain damage included the cerebellum (Table 1).

### 2.2. Paradigm

**Figure 1:**
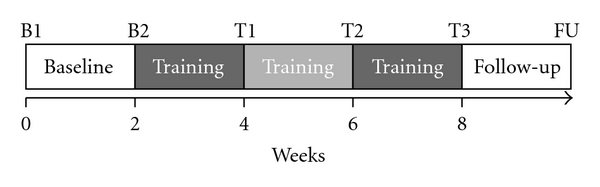
Timeline of climbing training and motor performance tests. B1, B2: baseline tests before training. T1, T2, T3: tests during and at the end of the training period. FU: follow-up test after training.

The patients performed a 6-week climbing training. The effect of this training on ataxia was evaluated with unrestricted 3-dimensional arm and leg pointing movements, balance and manual dexterity tests and a questionnaire on self-perception of symptoms. The pointing movements and the balance and manual dexterity tests were performed 6 times at intervals of 2 weeks: before (B1, B2), during (T1, T2, T3), and after the training period (FU) (Figure 1). The questionnaire was completed 6 times, at the end of each training week. 

### 2.3. Procedure

#### 2.3.1. Climbing Training

The training took place on 2 climbing walls, which were 2.5 m high. The inclination of one wall was adjustable from 0° to 45°, whereas the second wall was almost vertical with a structured rough surface. Standard climbing equipment was available to secure the patients. The frequency and duration of the training sessions were scheduled taking into consideration the state of health and physical condition of each patient (Table 2). The goal was to train as much as possible avoiding overtraining. The climbing exercises were prepared individually with the aim to maximally challenge each patient's main motor deficits. For patient 1, particular emphasis was put into the coordination of the limbs and the speed of single movements as well as movement sequences. For patient 2, the main goal was to improve movement accuracy and balance. For patient 3 the main focus lay in the appropriate turning of the head and the integration of visual information in the movement strategy. Patient 4 performed complex movement tasks that demanded concentration and physical endurance. Climbing exercises were prepared as manifold as possible in order to facilitate a transfer of learned motor patterns to everyday life and to keep the training interesting for the patients. The various exercises challenged body balance (e.g., using only specific grips, climbing very slowly, climbing diagonally, and laterally); movement accuracy in pointing and grasping (e.g., pointing to the next grip, returning, and afterwards grasping the next grip as accurately as possible); movement smoothness (e.g., climbing while balancing balloons filled with rice); movement velocity (e.g., climbing as fast as possible); the planning of single movements and movement sequences (e.g., looking at a grip, closing eyes, and reaching the grip); the integration of somatosensory information; (e.g., climbing with closed eyes). The patients rested whenever they felt tired. Once they were able to perform a task fluently without mistakes, the level of difficulty was increased according to therapist and patient preferences/goals (e.g., increased speed, closed instead of open eyes, and less body support). The goal was to challenge the patients as much as possible without exhausting them.

#### 2.3.2. Pointing Task

The patients used their clinically more affected right arm and leg to perform fast and accurate pointing movements.

**Arm Pointing Movements**

They were seated at a table with the height of the chair adjusted such that when the arms rested on the table, the forearms were parallel with the table surface. The chair was pushed towards the table until the subject's chest was in contact with the table, in order to prevent trunk motion while pointing to the targets. However, although the distance of the subject to the table was such that all targets could be comfortably reached by the index finger without trunk movements, it cannot be excluded that small trunk movements occurred. Four ball targets of a diameter of 2 cm, labelled with the numbers 1 to 4 were suspended from a fine wire above a table in a semicircle around 2 starting points. The starting points, labelled with colours, were right of the subject's midline ipsilateral of the performing arm (13 cm from the edge of the table) and left of the subject's midline on the contralateral side (34 cm from the edge of the table) such that they could be comfortably reached. The target positions varied in height (5–35 cm) above the table and horizontal distance to the starting points (14–48 cm), which resulted in 8 different movement paths.

**Leg Pointing Movements**

The subjects stood upright and held a walking aid with both hands, providing body stability (patient 2, who was wheelchair-bound, was seated on a walking aid with both legs in the air, at a distance to the targets which allowed him to reach them comfortably). They performed pointing movements with their right big toe from one starting point towards 4 target balls resulting in 4 different movement paths. The 4 target balls (4 cm in diameter) were suspended above the ground in a semicircle around the starting point. Their positions varied in height above the ground (13–32 cm) and in horizontal distance to the starting point (25–36 cm).

Two seconds after the investigator had specified the starting point (had to be announced only for arm movements) and the target, an acoustic “go-signal” was given, upon which the subjects had to touch as fast and accurately as possible the middle of the target ball with the tip of their right index finger, respectively, right big toe and to return to the starting point. The targets and starting points were announced in pseudorandom order. In order to be sure that the subject understood the instructions, the pointing movements were practiced 1–5 times before the measurements started. Each subject performed at least 24 (6 movements/target) up to 80 (20 movements/target) arm movements and 12 (3 movements/target) up to 40 (10 movements/target) leg pointing movements.

The 3-dimensional movement paths were recorded with a motion measuring system (CMS30 P, Zebris Medizintechnik GmbH, Isny) consisting of miniature ultrasound transmitters, a measuring sensor with 3 ultrasound microphones, and the basic unit. One transmitter was fixed on the tip of the right index finger and another one on the tip of the right big toe. The basic unit computed the position of the ultrasound transmitters and recorded data from an acoustic signal generator (generating “go-signals” for the pointing task).

#### 2.3.3. Clinical Balance and Motor Skill Tests

The patient's balance and manual dexterity was evaluated using the 2 following clinical tests.

**(1) Berg Balance Test [14]**

The patients were asked to perform 14 different functional activities such as standing unsupported, reaching forward while standing, turning 360°, and retrieving objects from the floor. A 5-point ordinal scale from 0 to 4, with 4 as the maximal possible score, quantified the performance.

**(2) Box and Block Test of Manual Dexterity [15]**

A box, divided into 2 equal sections, contained about 100 wooden cubes of 2.5 cm in the section ipsilateral to the tested hand. The patients were instructed to transfer as many blocks as possible to the other section, grasping one block at a time. The test was performed with the right as well as with the left hand. The number of blocks transported to the other section during 60 s was the measure of performance.

#### 2.3.4. Self-Perception of Motor Symptoms

The questionnaire addressed the physical activities besides the climbing training, the motivation for the climbing training, movement control in general (e.g., during the performance of slow movement sequences), and the performance in certain everyday skills which were defined before the start of the study for every patient individually (e.g., brushing teeth, cutting food, or tying shoes). Answers were given both in writing and with the help of a 5-point ordinal scale.

### 2.4. Data Analysis

The position data of the 3-dimensional pointing movements and the digital data encoding the time of the acoustic “go-signals” were recorded with the WinData software (Zebris Medizinaltechnik GmbH, Isny) and analyzed with programs developed in LabView (National Instruments, Austin, Tex, USA). Only correctly recorded (not more than 3 missing values and no accelerations greater than 25 m/s^2^) and correctly performed trials (no movement starts before the go-signal, and no aiming in a wrong direction) were analyzed. The following movement parameters were calculated:

**Movement Velocity MVel (m/s)**

Straight line from the starting point till reaching the target and back to the starting point divided by the elapsed time as a measure for how fast the movement was executed.

**Peak Speed PS (m/s)**

Maximal movement velocity between beginning the movement and the reaching of the target.

**Symmetry Index SI**

Time interval between the beginning of the movement and the peak speed, divided by the time interval between the beginning of the movement and reaching the target.

**End-Point Error EE (cm)**

Distance between the actual target position and the position of the subject's limb, when it was supposed to reach the target.

**Path Length PL**

Length of the movement path between the beginning of the movement and reaching the target divided by the length of a straight line between these 2 markers.

**Direction Changes DCh (s^−1^)**

Number of direction changes between the beginning of the movement and reaching the target divided by the time elapsed between these 2 markers, in order to avoid an influence of path length and movement velocity.

Outliers were identified based on interquartile range (IQR) (±10 IQR, <3% excluded). To assess the performance changes of the pointing movements for the patient group as a whole we performed for each movement parameter and experimental condition (arm, leg) a mixed model ANOVA with “test” (BL (mean of B1 and B2), T1, T2, T3, FU) as fixed factor and “subject” (P1, P2, P3, P4) as random factor, and a priori tests for differences between baseline (BL) and subsequent test sessions (T1, T2, T3, and FU). For each individual patient a one-way ANOVA was performed with “test” as fixed factor. However, we unfortunately did not have enough correctly recorded and performed trials at each test session to analyze the leg pointing movements of patient 2. For the same reason also the follow-up (FU) leg performance of patient 4 and the FU arm performance of patient 3 could not be analyzed.

For the clinical balance and motor skill tests, performance of each individual patient was evaluated by a visual display of the scores at each test session. Patients 2 and 3 could not be tested at FU. These data are therefore not displayed. The questionnaires on self-perception of symptoms were not always fully completed and thus only qualitatively analyzed.

## 3. Results

### 3.1. Pointing Movements

**Figure 2:**
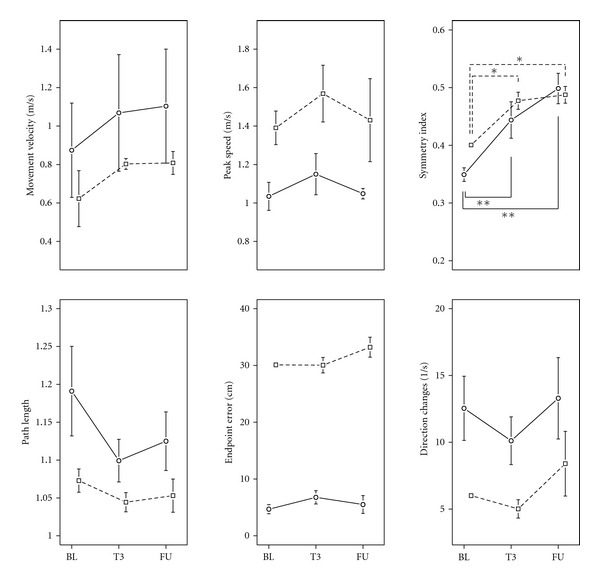
Pointing movements. Means ± 1 SE at baseline (BL, mean of B1 and B2), at the end of the training period (T3) and at follow-up 2 weeks after the end of the training period (FU). For the symmetry index performance at T3 and FU was significantly different from performance at BL for both arm and leg movements. *P < 0.05,**P < 0.001. Arm movements: solid line, leg movements, dashed line.

Overall, in the course of the climbing training the movements of the patients seemed to become faster (increase of MVel and PS), smoother (decrease in amount of DCh), and less curved (decrease of PL). Furthermore; the speed profile became more symmetrical (increase of SI) (Figure 2). However, there was a significant main effect of “test” on the SI only (arm: *F*(4, 20.82) = 8.79, *P* < 0.001; leg: *F*(4, 11.83) = 4.03, *P* < 0.05). A priori comparisons revealed a significant difference to BL at T2 (*P* < 0.05), T3 (*P* < 0.001), and FU (*P* < 0.001) for the arm movements and at T1 (*P* < 0.001), T3 (*P* < 0.01), and FU (*P* < 0.05) for the leg movements (Figure 2).

 The individual analysis of the patients revealed that out of a total of 42 parameters; 19 significantly changed in the direction of improved motor performance, 3 towards deteriorated performance, and 2 improved or deteriorated depending on the test session (Table 3). Patient 1 significantly improved in 6 and got worse in one parameter out of 12, patient 2 improved in 3 and deteriorated in one out of 6 parameters, patient 3 improved in 3 and deteriorated in one out of 12 parameters, and patient 4 improved in 7 parameters out of 12. MVel and SI were the parameters showing the most significant improvements (MVel: 6 out of 7, SI: 5 out of 7) and in no patient a deterioration of performance was observed.

### 3.2. Balance and Motor Dexterity Tests

**Box and Block Test of Manual Dexterity**

**Figure 3:**
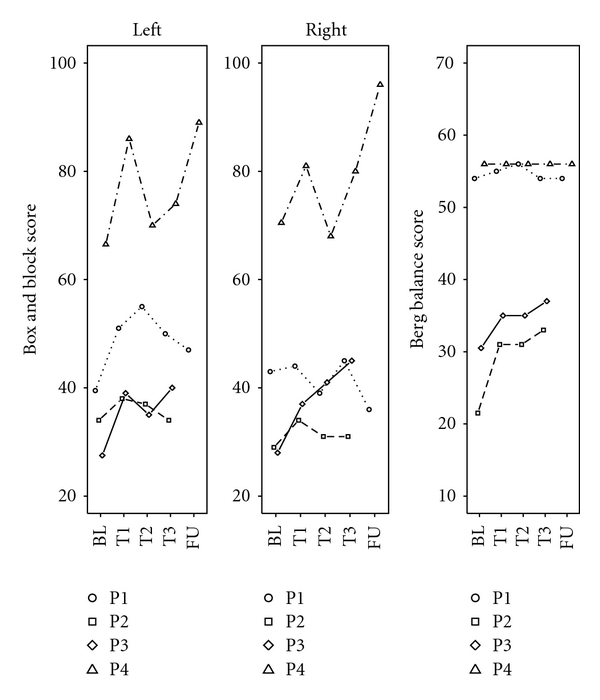
Manual dexterity and balance. Box and block scores and Berg balance scores for each patient. FU for patients 2 and 3 not displayed (see Section 2.4).

Patients 3 and 4 improved their performance for the left as well as for the right hand (P3 left: 27.5 to 40, right: 28 to 45; P4, left: 66.5 to 89, right: 70.5 to 96). Patient 1 improved only for the left hand (left: 39.5 to 47, right: 43 to 36), while the performance of patient 2 remained more or less stable (left: 34 to 34, right: 29 to 31, see Figure 3).

**Berg Balance Test**

Figure 3 shows that patients 2 and 3 improved from BL to T3 at the end of the climbing training (P2: 21.5 to 33, P3: 30.5 to 37, out of a maximal score of 56), whereas the performance of patients 1 and 4 remained unchanged from BL to FU, which was expected, since these patients already performed at a very high level before the training (P1: 54 to 54, P4: 56 to 56, out of a maximal score of 56).

### 3.3. Self-Perception of Motor Control

**Patient 1**

He reported a reduction of tremor and an improvement in handling cups and tying shoes. This patient's motivation for the climbing training was moderate to good. He liked climbing as a welcome change in everyday life.

**Patient 2**

The patient expressed a feeling of greater body stability and gained confidence in his capacity to control equilibrium and therefore dared more to shift his body weight to a single leg during locomotion. He also reported constantly improving accuracy and higher velocity of his right arm and leg movements in everyday life. In the second half of the training period, he noticed slightly smoother movement paths of his right arm and leg and a slight increase in speed when performing movement sequences. In the second and third weeks, however, he felt a temporary increase of tremor. This patient was always very motivated and appreciated the physical activity, especially the upright body position during climbing as a welcome change to the sitting position in the wheelchair.

**Patient 3**

The climbing training had a positive effect on body position and stability and on the use of his visual system, that is, on the appropriate turning of his head towards movement targets. The patient also noticed a slight increase in speed of single movements and movement sequences.

**Patient 4**

After a temporary deterioration in the second training week, the patient reported an improved ability to perform slow movement sequences and a decrease in tremor. Slow movements, performed without concentration, became smoother from the third week onwards. Rapid movements became faster and improved constantly in accuracy from the fourth week until the end of the training period. The patient liked climbing as a physical activity and was always very motivated.

## 4. Discussion

The aim of this study was to investigate whether climbing training is a suitable treatment for patients with cerebellar disorder. The 4 patients who participated in this study were very different in terms of pathology. Nevertheless all of them did improve in several measures for movement quality and hardly deterioration of motor control was detected. They also reported improvements of movement quality in daily life.

As expected there was a high intersubject variability in the motor improvements in the course of the climbing training, very likely due to the differences in pathology, duration of illness [16] and probably other factors such as age and motivation. However, even though the parameters that improved differed from patient to patient, all patients were able to improve their performance in fast and accurate multijoint pointing movements. In the course of climbing training, the patient group as a whole, achieved a more symmetrical movement speed profile in arm as well as in leg movements. Since movement accuracy did not decrease, we assume that there was no change in movement strategy but a real improvement of motor performance.

Furthermore, patients 2 and 3, who suffered from a severe balance disturbance, improved their whole body balance, as assessed by the Berg balance test. This supports previous findings that balance and locomotor training improves postural stability and gait in patients with cerebellar dysfunction [6, 17, 18]. The other patients had no balance disturbances initially and therefore no potential to improve. Patients 3 and 4 improved manual dexterity for the left and the right hand, as assessed by the box and block test.

Overall our data support the suggestion that long-term coordinative training improves motor performance and reduces ataxia symptoms in patients with cerebellar ataxia [19, 20]. Furthermore, the fact that improvements occurred in upper as well as in lower limbs goes in line with our suggestion that climbing training is a suitable method to train the whole motor system. For patient 3, cognitive impairment, including attention deficits and impairments within the visual system, might explain why he improved motor performance less than the other patients.

Although we suggest that patients with cerebellar disorder are still able to learn, they might use a different learning strategy. Discussions with the patients and observations during the climbing training indicated that the performance of complex movement tasks often caused difficulties. According to reports of 2 patients this might have been at least partly due to the fact that they had to coordinate their movements rather consciously. This is in line with previous findings that the automaticity of movements and implicit learning are disturbed in patients with cerebellar disorder [2, 21]. However, as shown in this study, this phenomenon does not seem to impede an improvement of motor control. 

Limitations of this study are the small number of subjects, and that we could not test a control group of patients without climbing training. We can, therefore, not prove that the observed motor improvements are exclusively due to the climbing training. However, we observed during the climbing training that the patients' movement velocity increased, movement paths became smoother, and fewer corrections were required to reach the climbing grips, when a task was practiced several times in succession within a training session. This circumstance, as well as the fact that the patients themselves reported improvements in their everyday movements, speaks for a more integral improvement of motor control, not only due to the learning of the motor tests. We can also not exclude an effect of target position or trunk movements, since our study was not designed to assess the influence of these factors. Our participants were, however, seated with their chest in touch with the table edge in order to minimize trunk movements, and we did not observe increasing trunk movements across test sessions.

In conclusion, our data provides evidence that climbing training might have a positive effect on the coordination of upper and lower limb movements and on balance and may thus have therapeutic potential.

## Figures and Tables

**Table 1:** Patient characteristics; CT: computed tomography; FAC: functional ambulatory category [13].

Patient	Age	Diagnosis	Lesion (CT)	Duration of illness	Mobility FAC 0–5	Other symptoms
1	29	Craniocerebral injury	Diffuse axonal injury with bilateral frontal, occipital, and hippocampic hemorrhagic lesions with hydrocephalus	2 years	4	—

2	56	Ischemic lacunar stroke, sequelae of left vertebral aneurysm with ventriculocardiac derivation	Sequelae of clipping, no acute lesion	2 months (stroke) 25 years (aneurysm)	1	Right sensitive-motor hemisyndrome

3	22	Perinatal anoxic encephalopathy	Intraventricular haemorrhage	22 years	4	Psychomotor retardation, oculomotor signs

4	42	Metabolic encephalopathy with epilepsy after stereotactic biopsy of a frontal lobe tumor (3.5 years before study onset) and subsequent chemotherapy	Left frontal expansive lesion of corpus callosum with extension to the left temporal lobe	8 months	5	Hypokinetic movement bilateral

**Table 2:** Training schedule for each patient from weeks (w) 1 to 6.

	w1	w2	w3	w4	w5	w6
P1	2 × 45 min	2 × 45 min	2 × 45 min	3 × 45 min	3 × 45 min	3 × 45 min
P2	2 × 30 min	2 × 40 min	2 × 40 min	2 × 40 min	3 × 40 min	3 × 40 min
P3	2 × 45 min	2 × 45 min	2 × 45 min	3 × 45 min	3 × 45 min	3 × 45 min
P4	3 × 60 min	3 × 60 min	3 × 60 min	3 × 60 min	3 × 60 min	3 × 60 min

**Table 3:** Significant performance changes compared to baseline (BL). Significant improvements of motor performance in bold, deterioration of performance in italic, ns: not significant. BL: mean of B1 and B2, baseline tests before training. T1, T2, T3: tests during and at the end of the training period. FU: follow-up test after training.

		Movement velocity (m/s)	Peak speed (m/s)	Symmetry index	Path length	Endpoint error (cm)	Direction changes (s^−1^)
P1	Arm	**T3, FU**	ns	**T2, T3, FU**	*T1, FU*	ns	ns
	Leg	**T1, T2, T3, FU**	ns	**T1, T2, T3, FU**	**T1, T2, T3, FU**	ns	**T1, T2, T3, FU**

P2	Arm	**T1, T2, T3, FU**	**T1, T3**	**FU**	ns	*T2, T3, FU*	ns

P3	Arm	ns	ns	ns	**T1, T2, T3**	ns	ns
	Leg	**T3, FU**	**T3, FU**	ns	ns	ns	*T1*

P4	Arm	**T2, T3, FU**	ns	**T2, FU**	**T1, T2, T3, FU**	**T2, ** *T3, * **FU**	**T1**, *T2 *
	Leg	**T1, T2**	**T2**	**T1, T2**	ns	ns	**T1**
