# Erratum to “Effect of Long-Term Climbing Training on Cerebellar Ataxia: A Case Series”

**DOI:** 10.1155/2014/207137

**Published:** 2014-02-03

**Authors:** Marianne Anke Stephan, Sylvie Krattinger, Jérôme Pasquier, Shahid Bashir, Thomas Fournier, Dieter Georg Ruegg, Karin Diserens

**Affiliations:** ^1^Department of Medicine, University of Fribourg, 1700 Fribourg, Switzerland; ^2^Neurological Center Plein Soleil, 1010 Lausanne, Switzerland; ^3^Department of Mathematics, University of Fribourg, 1700 Fribourg, Switzerland; ^4^Unit of Early Neurorehabilitation, Department of Clinical Neurosciences, University Hospital, 1011 Lausanne, Switzerland

The authors' names were incorrectly listed as Stephan Marianne Anke, Krattinger Sylvie, Pasquier Jérôme, Bashir Shahid, Fournier Thomas, Ruegg Dieter Georg, and Diserens Karin; this error is corrected here.

